# The impact of school life and holidays on sleep and physical activity patterns in junior high school students: a small-sample longitudinal study using wearable devices

**DOI:** 10.3389/fpubh.2026.1833198

**Published:** 2026-06-01

**Authors:** Yun Chen, Kai Xu, Yifan Zhao, Zhongke Gu, Shanshan Zhang, Gangrui Chen, Jiansong Dai

**Affiliations:** 1 Department of Sport and Health Sciences, Nanjing Sport Institute, Nanjing, China; 2 High School Affiliated to Nanjing Normal University Xianlin Department Of Middle School, Nanjing, China; 3 Sport Science Research Institute, Nanjing Sport Institute, Nanjing, China

**Keywords:** adolescents, physical activity, sleep, structured day hypothesis, wearable devices

## Abstract

**Background:**

The structured day hypothesis posits that the characteristics of a structured school environment play a protective role in adolescents’ physical activity. Existing studies predominantly rely on subjective reports or short-term monitoring, lacking longitudinal objective evidence that covers a full academic year cycle for Chinese students who are under high academic pressure.

**Objective:**

This study employs long-term objective monitoring through wearable devices to systematically compare sleep and physical activity patterns among Chinese middle school students across four phases: study days, weekend days, winter vacation, and summer vacation, while also analyzing gender differences.

**Methods:**

A longitudinal study was conducted involving 27 first-year middle school students (14 boys and 13 girls) from a middle school in Nanjing. The Huawei Band 6 was utilized to continuously monitor sleep parameters, including sleep onset time, wake-up time, duration of deep, light, and REM sleep, as well as total sleep duration. Additionally, physical activity parameters such as step count, MVPA, and continuous MVPA were recorded during the specified four stages. A linear mixed-effects model was employed to analyze the effects of both stage and gender.

**Results:**

On school days, insufficient sleep was observed alongside relatively high levels of physical activity, with only 26.71% of participants meeting the sleep adequacy standard and 34.30% achieving the MVPA target. During holidays, sleep quality improved while physical activity levels decreased, with the MVPA compliance rate among girls dropping to a mere 3.83% on weekend days. In contrast, winter vacation showed significantly higher total sleep duration, deep sleep duration, and step counts compared to summer vacation. Notably, girls exhibited longer deep sleep duration than boys; however, their step counts and MVPA levels were significantly lower than those of boys.

**Conclusion:**

Structured school schedules promote the maintenance of physical activity; however, they may also lead to increased sleep deprivation. In contrast, holidays tend to improve sleep quality but are linked to a significant decline in physical activity levels. Therefore, it is imperative to optimize daily routines during school days to ensure adequate sleep. Furthermore, establishing a collaborative intervention mechanism that involves families, schools, and communities during holidays can encourage physical activity and reduce sedentary behavior and screen time, particularly among female students and during summer vacations.

## 1 Introduction

Adequate sleep and regular physical activity are fundamental pillars that ensure the physical health, cognitive development, and mental well-being of adolescents (1). Research indicates that sleep plays a critical role in the physiological growth, nervous system function, and immune response of adolescents, while also influencing cognitive performance, academic achievement, and emotional regulation (2–4). Sleep deprivation can result in decreased attention (5), poorer academic performance (6), and increased risks of psychological issues such as anxiety and depression (7). In terms of physical activity, moderate-to-vigorous physical activity (MVPA) is recognized as a crucial factor in promoting cardiovascular function, bone health, and metabolic balance (8). Furthermore, it helps alleviate stress, improve emotional states, and enhance quality of life (9, 10).

The World Health Organization recommends that children and adolescents aged 5–17 accumulate at least 60 min of moderate-to-vigorous physical activity daily to promote both physical and mental health (11). Additionally, the American Academy of Sleep Medicine advises that adolescents should aim for 8–10 h of sleep each night to ensure optimal physiological and cognitive functioning (12). Despite these guidelines, the global adolescent population continues to face the dual health challenges of sleep deprivation and insufficient physical activity (13, 14), a situation that is particularly pronounced in East Asia (15). In the high-pressure academic environment of China, adolescents typically endure significant academic workloads and stress (16). The demands of prolonged learning, frequent extracurricular tutoring, and rigid school schedules substantially reduce both their sleep duration and exercise time (17, 18).

The Structured Days Hypothesis (SDH) posits that the highly structured environment of schools imposes significant constraints on adolescents’ daily behaviors through fixed schedules and curricula (19). During the school semester, early wake-up times for school attendance may limit sleep duration, whereas physical education classes, recess activities, and commuting behaviors may partially maintain physical activity levels (20, 21). In contrast, the less structured environment of holidays increases time autonomy but may lead to delayed sleep schedules, disrupted circadian rhythms, and significant increases in screen time and sedentary behaviors (22). Current research on holidays continues to reveal notable controversies.

Previous research on adolescent sleep and physical activity has certain methodological limitations (23). Firstly, traditional studies predominantly rely on questionnaire surveys or retrospective self-reports (24). There remains a scarcity of longitudinal behavioral monitoring evidence that encompasses complete academic year cycles, including study days, weekend days, and winter/summer vacations, among Chinese junior high school students. Particularly in the context of China’s high academic pressure environment, the characteristics of adolescents’ sleep architecture and physical activity patterns, along with the associated issues, warrant further investigation.

Based on the shortcomings of previous research, this study employs objective wearable devices to conduct longitudinal monitoring of junior high school students in the developed regions of Eastern China over an entire academic year. The aim is to utilize wearable technology to systematically compare the characteristics of sleep structure and physical activity patterns across four phases of the academic year: school weekdays, school weekends, winter vacation, and summer vacation, while also conducting an in-depth analysis of gender differences. This will reveal the patterns of time allocation and behavioral pathways of adolescents during the complete academic year in a high academic pressure environment in developed regions of Eastern China, providing a scientific basis for optimizing educational policies and managing family health during holidays.

## 2 Methods

### 2.1 Study subjects

The sample size was estimated using G*Power software (version 3.1.9.7) with repeated measures ANOVA as the calculation method. Based on previous studies (25) and Cohen’s statistical criteria (26), the effect size was set at *f* = 0.3 (medium effect), with a power of 0.80, significance level *α* = 0.05, and an intra-group repeated measures correlation coefficient of 0.5. The calculation indicated that the minimum effective sample size was 20 participants. Considering a potential 20–30% loss to follow-up or invalid data among students, the final sample size was determined to be 30 participants. The study subjects were selected from all first-year junior high school students at Xianlin School Affiliated to Nanjing Normal University in Nanjing, Jiangsu Province. Using a random cluster sampling method, four classes were randomly selected from the 10 classes of first-year junior high school students. Recruitment was conducted among students from these four classes and their legal guardians, resulting in a total of 30 students included in the study. During the research process, three participants were excluded due to incomplete data collection or failure to meet wearing requirements, resulting in 27 valid samples (14 boys and 13 girls) and a loss-to-follow-up rate of 10%. As Nanjing is a representative city in China’s developed eastern region, the findings of this study can, to some extent, reflect the sleep and physical activity patterns of junior high school students in developed areas of China. The basic characteristics of the participants are described in Table 1. The inclusion criteria were as follows: (1) good physical health without limb disabilities; (2) no contraindications to exercise; (3) no clinically diagnosed mental or psychological disorders. This study strictly adhered to the ethical guidelines for human subject research outlined in the Declaration of Helsinki and was approved by the Human Research Ethics Review Committee of Nanjing Sport Institute (Approval No.: RT-2021-02). Prior to the commencement of the study, the researchers provided detailed explanations of the research objectives, procedures, and potential risks to the school administration, teachers, and parents. All participants and their legal guardians were fully informed of the study content and provided written informed consent.

**Table 1:** Basic information of participants.

Characteristics	Males (*n* = 14)	Females (*n* = 13)
Age (years)	12.94 ± 0.30	12.89 ± 0.29
Height (cm)	164.93 ± 6.27	159.19 ± 5.51
Weight (kg)	56.79 ± 10.60	49.19 ± 9.65
BMI	20.85 ± 3.72	19.28 ± 2.78

Data are expressed as mean ± standard deviation (mean ± SD); BMI: body mass index.

### 2.2 Research methodology

The data encompassed three key phases with the following specifics: (1) during the school term: From December 7 to 20, 2021, a two-week data collection was conducted, yielding an average of 13.30 ± 1.14 valid wearing days. (2) Winter vacation period: From January 19 to February 13, 2022, the average number of valid wearing days was 19.00 ± 6.87. (3) Summer vacation period: From June 29 to August 30, 2022, the average number of valid wearing days was 24.59 ± 9.73. Given that the autumn and spring semesters exhibited a high degree of consistency in school schedules, students’ routines during the school term were primarily influenced by the institutional schedule structure rather than seasonal factors. Consequently, this study selected the autumn semester as representative of the in-semester phase, avoiding redundant data collection for the spring semester. This study was conducted during the normalization period of COVID-19 prevention and control in China. Due to the strict epidemic prevention measures in the country, there were no large-scale outbreaks in the schools and cities during the research period, and no lockdown management was implemented. The daily life order of students both on and off campus remained stable and orderly. These conditions ensured the smooth progress of this research, and there were no disruptions to the research process caused by the epidemic.

During the research phase, students strictly adhered to a 24-h continuous wear protocol, except for necessary removal situations such as bathing and charging. Researchers monitored backend data daily and reminded parents to upload data in a timely manner to ensure effective wear duration. In this study, a daily effective wear time of 16 h or more (≥16 h/day) is required for it to be considered effective wear and included in the statistical analysis. During the winter and summer breaks, after fulfilling the requirement of at least 14 days of device wear, students could voluntarily choose whether to continue wearing the device until the start of the new school term. Considering the differences in average wear days between school and vacation periods, this study employed the intra-class correlation coefficient (ICC) to conduct consistency checks on the sleep and physical activity parameters for each subject’s data from the first 14 days of each phase against their data during the complete wear period. The results indicated that the ICC values for all parameters were greater than 0.85, suggesting a high consistency between the data from the first 14 days and the data from the complete wear period, indicating that differences in wear days across different phases did not introduce systematic bias in the group behavior patterns.

This study employs the Huawei Band 6 (Huawei Band 6, Shenzhen, China), which integrates multiple sensors and algorithms to comprehensively monitor users’ physiological and exercise states. The device utilizes a Photoplethysmography (PPG) sensor to detect autonomic activities such as step count, heart rate, and pulse wave (27). The body movement signals collected by the accelerometer, in conjunction with machine learning algorithms (28), are utilized to measure sleep and wake states, with data transmission and management facilitated through the Huawei Research cloud platform and a dedicated mobile application. By applying Cardiopulmonary Coupling (CPC) technology and Heart Rate Variability (HRV) analysis, the accuracy of sleep staging and quality assessment has been enhanced (29, 30). This device has been utilized in physical activity research among adolescents (31), as well as in sleep studies involving female medical staff (32), athletes (33), and adolescents (34). Previous research has validated the good reliability and validity of this device in sleep assessment through comparisons synchronized with laboratory data (35).

### 2.3 Parameter definitions

This study extracted the following key parameters from the research platform:

(1) Sleep Characteristic Parameters: The sleep characteristic parameters include sleep onset time, wake-up time, duration of deep sleep, duration of light sleep, REM duration, wake duration, total sleep duration, daytime sleep duration, and nighttime sleep duration. Among these parameters, nighttime sleep duration is defined as the interval from the onset of sleep at night to the final awakening time the following day.

(2) Physical Activity Parameters: The Huawei wristband collects minute-by-minute heart rate data and step counts (36). To quantify exercise intensity per minute, the Heart Rate Reserve (HRR) method was combined with the Karvonen approach (37). The calculation formula is as follows: HRR = HRmax – HRrest, where the maximum heart rate (HRmax) is calculated using the Tanaka formula: HRmax = 208–0.7 × age (38). The resting heart rate (HRrest) is determined by selecting the heart rate during the 5 min before and after waking, as this state is least affected by external environmental factors and psychological stress (34). The relative exercise intensity per minute is expressed as a percentage of heart rate reserve, calculated using the formula: %HRR = (HR − HRrest)/(HRmax − HRrest) × 100%. This approach helps to mitigate the impact of individual heart rate differences on the determination of exercise intensity.

(3) Intensity Threshold and Classification: According to the guidelines set forth by the American College of Sports Medicine (ACSM) (39), minutes during which the %HRR was equal to or greater than 40% were classified as moderate-to-vigorous physical activity (MVPA). Continuous MVPA is defined as activity bouts that meet the MVPA intensity threshold and are sustained for at least 10 consecutive minutes.

(4) Time period division: According to the school calendar, weekdays (Monday to Friday) during the academic term are designated as “school days,” while Saturdays and Sundays are classified as “weekend days.”

### 2.4 Statistical analysis

Statistical analysis was conducted using JMP Pro 17 software (JMP Statistical Discovery LLC, Cary, NC, United States). First, the Shapiro–Wilk test was used to assess the normality of continuous variables. Continuous variables that conform to a normal distribution are expressed as “mean ± standard deviation,” along with the 95% confidence interval (95% CI). To avoid data errors, the individual mean of each parameter for each student was calculated before determining the overall phase mean for the sample. A linear mixed-effects model was employed to analyze the effects of different phases and gender, with phase and gender treated as fixed effects and individual students as random effects, thereby controlling for the intra-individual correlation of repeated measures. In this study, no covariance structure was included when employing the mixed-effects model. Additionally, residual analysis was conducted for all indicators, and the results showed that all residual plots fell within a reasonable range, thereby validating the basic assumptions of the mixed model. To control for Type I error inflation due to multiple comparisons, the Tukey HSD method was used for pairwise comparisons among all groups. This method provides a more rigorous basis for statistical significance determination in multiple group comparisons by controlling the family-wise error rate. Effect sizes were reported using ηp^2^, with thresholds of 0.01, 0.06, and 0.14 representing small, medium, and large effect sizes, respectively.

## 3 Results

### 3.1 Changes in sleep characteristics and physical activity patterns across different stages

**Figure 1:**
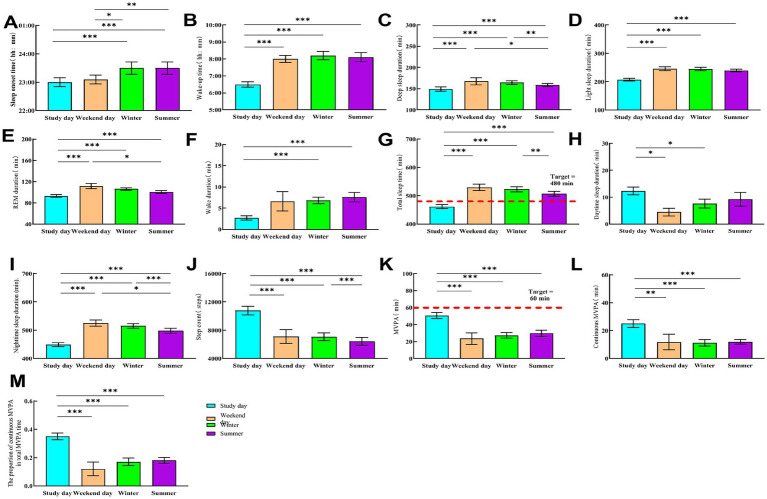
Comparison of sleep and physical activity at different stage. **(A)** Sleep onset time (hh:mm), **(B)** wake-up time (hh:mm), **(C)** deep sleep duration (min), **(D)** light sleep duration (min), **(E)** REM duration (min), **(F)** wake duration (min), **(G)** total sleep duration (min), **(H)** daytime sleep duration (min), **(I)** nighttime sleep duration (min), **(J)** step count (steps), **(K)** MVPA (min), **(L)** continuous MVPA duration (min), **(M)** the proportion of continuous MVPA duration to total MVPA duration. Red dashed horizontal lines indicate the targets for total sleep duration (480 min) and MVPA duration (60 min). *p < 0.05, **p < 0.01, ***p < 0.001.

Significant changes were observed in various sleep and physical activity parameters among junior high school students across different stages. The mixed-effects model revealed notable differences between stages in sleep onset time (*F* = 31.63, *P* < 0.0001, *η*_p_^2^ = 0.0603), wake-up time (*F* = 154.25, *P* < 0.0001, *η*_p_^2^ = 0.2384), deep sleep duration (*F* = 16.73, *P* < 0.0001, *η*_p_^2^ = 0.0333), light sleep duration (*F* = 62.17, *P* < 0.0001, *η*_p_^2^ = 0.1134), REM duration (*F* = 17.2, *P* < 0.0001, *η*_p_^2^ = 0.0341), wake duration (*F* = 11.05, *P* < 0.0001, *η*_p_^2^ = 0.0221), total sleep time (*F* = 65.55, *P* < 0.0001, *η*_p_^2^ = 0.1189), daytime sleep duration (*F* = 3.71, *p* = 0.0112, *η*_p_^2^ = 0.0076), nighttime sleep duration (*F* = 77.8, *P* < 0.0001, *η*_p_^2^ = 0.1380), step count (*F* = 78.69, *P* < 0.0001, *η*_p_^2^ = 0.1337), MVPA (*F* = 28.69, *P* < 0.0001, *η*_p_^2^ = 0.0538), continuous MVPA duration (*F* = 18.52, *P* < 0.0001, *η*_p_^2^ = 0.0353), and the proportion of continuous MVPA in total MVPA time (*F* = 31.40, *P* < 0.0001, *η*_p_^2^ = 0.0620). Total sleep duration, REM duration, and deep sleep duration on weekend days, winter vacations, and summer vacations were significantly higher than on school days. The highest proportion of students meeting sleep standards occurred on weekend days, with 80% achieving the recommended 8 h of total sleep time; in contrast, this proportion on school days was only 26.71% (*P* < 0.05). Conversely, step counts and exercise intensity were greater on school days compared to weekend days and vacations. However, the proportion of students meeting the recommended 60 min of moderate-to-vigorous physical activity per day on school days peaked at only 34.3%. A comparison between winter and summer vacations indicated that total sleep duration (*T* = 3.49, *p* = 0.0028), deep sleep duration (*T* = 3.16, *p* = 0.0087), and step count (*T* = 3.89, *p* = 0.0006) during winter vacation were significantly higher than those during summer vacation, as illustrated in Table 2 and Figure 1.

**Table 2:** Characteristics of sleep and physical activity changes across different stages.

Parameter	Study day	Weekend day	Winter	Summer
Sleep parameters				
Sleep onset time (hh:mm)	23:00 ± 0:35 (22:45,23:14)	23:09 ± 0:38 (22:53,23:24)	23:32 ± 0:42 (23:15,23:48)	23:35 ± 0:53^&^ (23:13,23:56)
Wake-up time (hh:mm)	6:31 ± 0:21 (6:23,6:40)	8:00 ± 1:01 (7:36,8:24)	8:13 ± 0:40 (7:58,8:29)	8:04 ± 0:51^&^ (7:44,8:24)
Deep sleep duration (min)	149.23 ± 26.37 (138.80,159.67)	167.45 ± 44.09 (150.01,184.89)	164.43 ± 22.64 (155.47,173.39)	158.63 ± 21.03^&^ (150.31,166.94)
Light sleep duration (min)	207.07 ± 23.45 (197.80,216.35)	245.65 ± 36.12 (231.37,259.94)	244.6 ± 30.63 (232.48,256.71)	238.95 ± 26.22^&^ (228.58,249.33)
REM duration (min)	92.87 ± 13.56 (87.51,98.24)	111.71 ± 26.6 (101.18,122.23)	106.21 ± 13.14 (101.01,111.41)	100.49 ± 14.54^&^ (94.74,106.24)
Wake duration (min)	2.7 ± 2.66 (1.65,3.75)	6.62 ± 11.74 (1.98,11.27)	6.82 ± 4.09 (5.20,8.44)	7.61 ± 5.9^&^ (5.27,9.94)
Total sleep duration (min)	461.5 ± 36.22 (447.18,475.83)	529.26 ± 57.33 (506.58,551.94)	522.82 ± 45.53 (504.81,540.83)	507.31 ± 44.79^&^ (489.59,525.03)
Daytime sleep duration (min)	12.32 ± 7.77 (9.25,15.40)	4.44 ± 7.19 (1.60,7.29)	7.58 ± 8.69 (4.15,11.02)	9.24 ± 13.25^&^ (4.00,14.48)
Nighttime sleep duration (min)	449.18 ± 36.27 (434.83,463.53)	524.81 ± 55.82 (502.73,546.90)	515.24 ± 41.86 (498.68,531.79)	498.07 ± 46.02^&^ (479.87,516.28)
Percentage of achieving 8-h sleep (%)	26.71	80	73.44	67.96
Physical activity parameters				
Step count (steps)	10759.44 ± 3122.87 (9524.08,11994.81)	7105.52 ± 5084.22 (5094.27,9116.77)	7077.52 ± 2952.30 (5909.63,8245.41)	6423.07 ± 2884.71^&^ (5281.92,7564.22)
MVPA (min)	50.65 ± 18.68 (43.26,58.04)	23.50 ± 35.76 (9.35,37.65)	27.35 ± 16.47 (20.84,33.87)	29.78 ± 18.60^&^ (22.42,37.14)
Proportion of MVPA reaching 60 min (%)	34.30	12.00	15.01	14.91
Continuous MVPA (min)	25.05 ± 14.13 (19.46,30.64)	11.78 ± 29.18 (0.23,23.32)	11.17 ± 11.70 (6.55,15.80)	11.89 ± 8.54^&^ (8.51,15.26)
Proportion of continuous MVPA in total MVPA time	0.35 ± 0.12 (0.31,0.40)	0.12 ± 0.25 (0.02,0.21)	0.17 ± 0.14 (0.11,0.22)	0.18 ± 0.11^&^ (0.13,0.22)

Data are expressed as Mean ± SD (95% CI); & indicates significant differences among the four phases: Study day, Weekend day, Winter, and summer (*P* < 0.05).

### 3.2 Longitudinal comparison of sleep and physical activity characteristics across different periods

**Figure 2:**
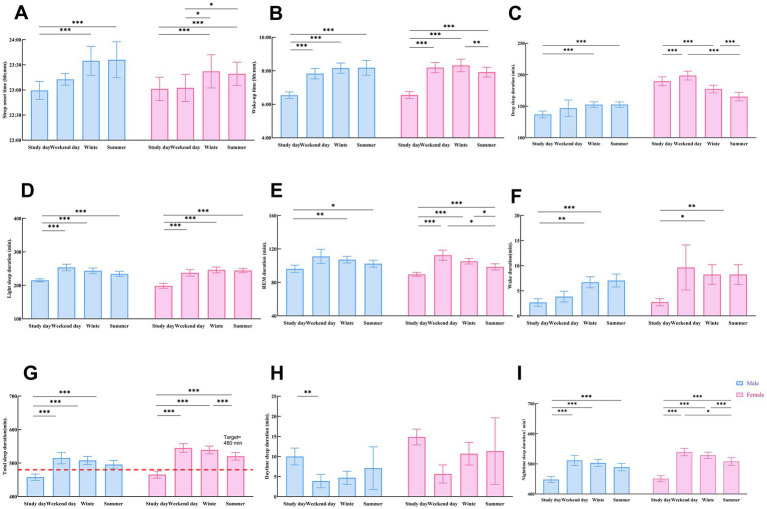
Changes in sleep parameters between males and females at different stages: **(A)** sleep onset time (hh:mm), **(B)** wake-up time (hh:mm), **(C)** deep sleep duration (min), **(D)** light sleep duration (min), **(E)** REM duration (min), **(F)** Wake duration (min), **(G)** total sleep duration (min), **(H)** daytime sleep duration (min), **(I)** nighttime sleep duration (min). In the total sleep duration plot, the red dashed horizontal line indicates the target sleep duration (480 min). *p < 0.05, **p < 0.01, ***p < 0.001.

**Figure 3:**
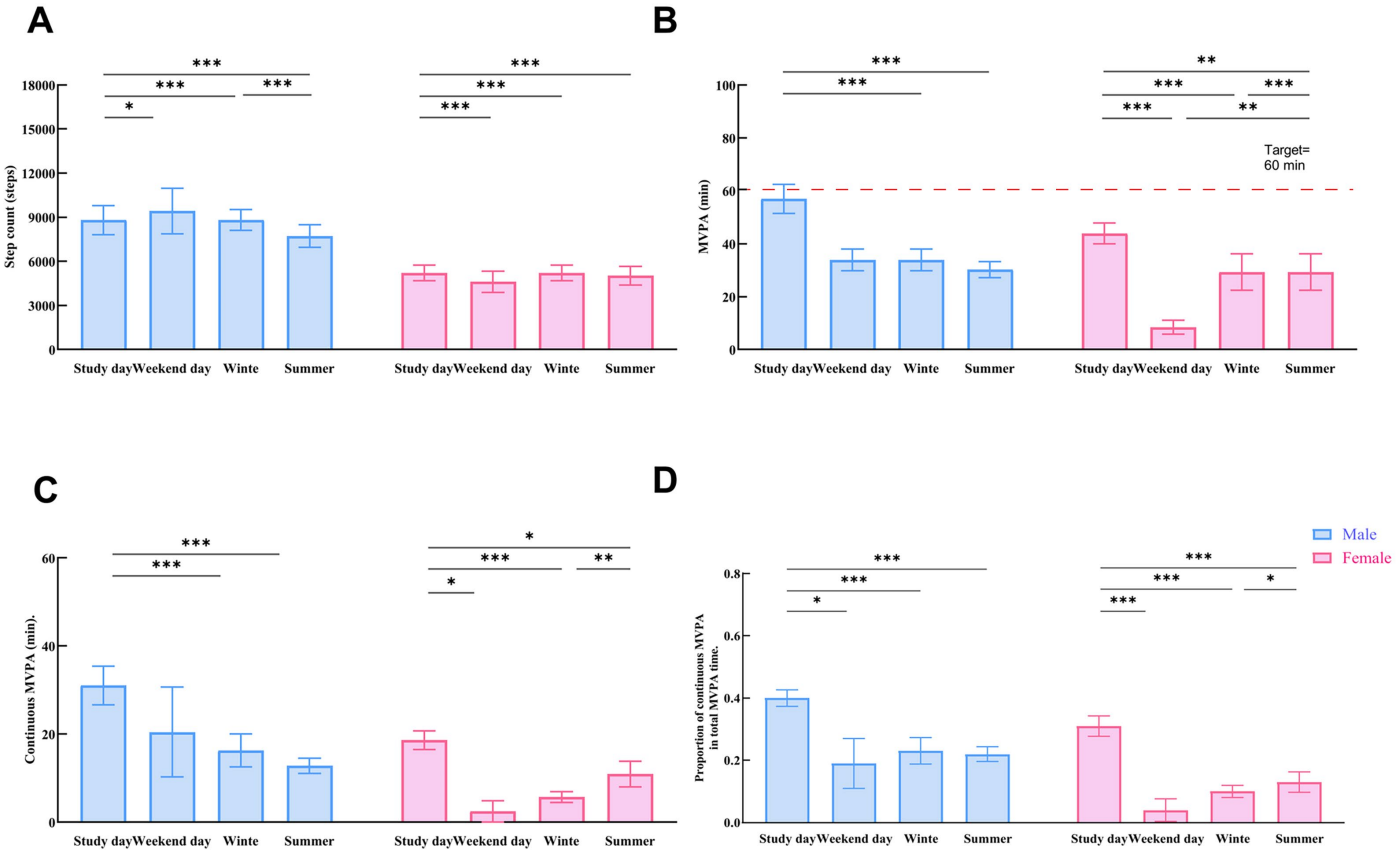
Changes in physical activity parameters between males and females at different stages: **(A)** Step count (steps), **(B)** MVPA (min), **(C)** duration of continuous MVPA (min), **(D)** proportion of continuous MVPA duration to total MVPA duration. In the MVPA plot, the red dashed horizontal line indicates the target MVPA duration (60 min). *p < 0.05, **p < 0.01, ***p < 0.001.

From a gender-stratified longitudinal comparison, the mixed-effects model analysis revealed consistent overall trends in sleep parameters between male and female students. All sleep metrics during school days were lower than those on weekend days, winter vacation, and summer vacation, with the exception of daytime sleep duration. The average total sleep time on school days was below 8 h, while it exceeded 8 h during other periods. Total sleep time (*T* = 3.96, *p* = 0.0005), deep sleep duration (*T* = 4.29, *p* = 0.0001), and REM duration (*T* = 2.69, *p* = 0.0363) during winter vacation were significantly higher than those during summer vacation. Regarding physical activity, both male and female students generally exhibited higher levels on school days compared to weekend days, winter vacation, and summer vacation. However, both boys and girls failed to meet the recommended daily standard of 60 min for moderate-to-vigorous physical activity (MVPA). Gender-specific differences were observed, with boys showing significantly higher step counts during winter vacation compared to summer vacation (*T* = 3.81, *p* = 0.0009). For girls, the intensity of moderate-to-vigorous physical activity (*T* = −3.88, *p* = 0.0007), continuous moderate-to-vigorous physical activity (*T* = −3.29, *p* = 0.0058), and the proportion of time spent in moderate-to-vigorous physical activity (*T* = −2.92, *p* = 0.0191) were all significantly lower during winter vacation than during summer vacation, as illustrated in Figures 2, 3.

### 3.3 Comparison of sleep and physical activity characteristics between male and female students at different stages

A comparison between males and females across different stages using mixed-effects models revealed that, in terms of sleep, females had significantly longer durations of deep sleep than males on school days (*F* = 7.98, *p* = 0.0093, *η*_p_^2^ = 0.2480), weekend days (*F* = 8.42, *p* = 0.0082, *η*_p_^2^ = 0.2741), and during winter vacation (*F* = 10.29, *p* = 0.0036, *η*_p_^2^ = 0.2899). No significant gender differences were observed in other sleep parameters (*p* > 0.05). Regarding sleep adequacy, females exhibited the highest proportion (96.15%) meeting the 8-h sleep target on weekend days, while the lowest proportion (26.71%) was recorded on school days. In terms of physical activity, male students had significantly higher step counts than female students on school days (*F* = 7.05, *p* = 0.0137, *η*_p_^2^ = 0.2220), weekend days (*F* = 7.64, *p* = 0.0109, *η*_p_^2^ = 0.2453), winter vacation (*F* = 15.06, *p* = 0.0006, *η*_p_^2^ = 0.3668), and summer vacation (*F* = 6.83, *p* = 0.0150, *η*_p_^2^ = 0.2166). Boys’ moderate-to-vigorous physical activity (MVPA) was significantly higher than that of girls on weekend days (*F* = 5.9, *p* = 0.0239, *η*_p_^2^ = 0.2138) and during winter vacation (*F* = 4.97, *p* = 0.0343, *η*_p_^2^ = 0.15508). Furthermore, boys exhibited higher levels of continuous MVPA and a greater proportion of total MVPA duration compared to girls on both school days and winter vacation (*P* < 0.05). Regarding MVPA compliance, the highest proportion of boys meeting the daily 60-min target was 40.63% on school days, whereas girls exhibited the lowest proportion of 3.83% on weekend days. This data is summarized in Table 3.

**Table 3:** Gender differences in sleep and activity across stages.

Parameter	Study day		Weekend day		Winter		Summer	
	Male	Female	Male	Female	Male	Female	Male	Female
Sleep parameters								
Sleep onset time (hh;mm)	22:59 ± 00:36 (22:42,23:17)	23:00 ± 00:42 (22:34,23:26)	23:15 ± 0:27 (22:59,23:30)	23:02 ± 0:48 (22:33,23:31)	23:33 ± 00:36 (23:12,23:54)	23:22 ± 01:11 (23:00,23:59)	23:43 ± 1:02 (23:07,0:19)	23:25 ± 00:43 (22:59,23:51)
Wake-up time (hh:mm)	06:30 ± 00:22 (6:17,6:43)	06:33 ± 00:21 (6:20,6:46)	7:50 ± 1:08 (7:10,8:29)	8:11 ± 0:54 (7:38,8:44)	08:03 ± 00:34 (7:43,8:23)	08:00 ± 01:19 (7:58,8:51)	08:05 ± 00:04 (7:28,8:42)	08:03 ± 00:36 (7:41,8:25)
Deep sleep duration (min)	136.85 ± 20.32 (125.11,148.58)	162.57 ± 26.23* (146.72,178.42)	146.88 ± 48.49 (118.88,174.87)	189.62 ± 25.27* (174.35,204.88)	152.54 ± 16.26 (143.15,161.93)	177.23 ± 21.95* (163.97,190.50)	152.6 ± 15.37 (143.44,161.76)	166.11 ± 22.93 (150.34,179.88)
Light sleep duration (min)	214.94 ± 16.75 (205.27,224.61)	198.86 ± 27.15 (182.19,215.01)	253.52 ± 35.96 (232.76,274.29)	237.18 ± 35.71 (215.60,258.76)	243.38 ± 31.36 (225.49,261.28)	245.91 ± 31.44 (226.90,264.90)	234.28 ± 25.46 (217.33,250.68)	244.29 ± 22.56 (230.43,258.14)
REM duration (min)	96.07 ± 16.45 (86.57,105.56)	89.43 ± 18.99 (84.00,94.86)	111.00 ± 31.36(92.90,129.10)	112.47 ± 21.61 (99.41,125.53)	107.11 ± 14.67 (98.64,115.58)	105.23 ± 11.80 (98.10,112.36)	102.35 ± 15.71 (93.28,111.42)	98.49 ± 13.51 (90.33,106.66)
Wake duration (min)	2.67 ± 2.84 (1.03,4.31)	2.74 ± 2.55 (1.19,4.28)	3.83 ± 4.01 (1.51,6.14)	9.63 ± 16.19 (−0.15,19.42)	6.69 ± 4.13 (4.30,9.07)	6.96 ± 4.21 (4.41,9.50)	7.03 ± 4.79 (4.27,9.80)	8.23 ± 7.06 (3.97,12.50)
Total sleep duration (min)	457.84 ± 34.88 (437.70,477.98)	465.45 ± 38.61 (442.12,488.78)	514.72 ± 64.23 (477.64,551.80)	544.91 ± 46.27 (516.95,572.87)	507.74 ± 44.79 (481.88,533.60)	539.06 ± 42.03 (513.67,564.46)	495.37 ± 46.43 (468.57,522.16)	520.18 ± 40.85 (495.50,544.86)
Daytime sleep duration (min)	9.99 ± 7.85 (5.45,14.52)	14.84 ± 13.01 (10.53,19.15)	3.32 ± 6.29 (−0.31,6.95)	5.65 ± 8.14 (0.73,10.57)	4.70 ± 6.08 (1.19,8.21)	10.69 ± 10.17 (4.55,16.84)	6.41 ± 10.58 (0.30,12.52)	11.29 ± 15.48 (2.93,21.64)
Nighttime sleep duration (min)	447.85 ± 37.29 (426.33,469.38)	450.61 ± 36.60 (428.49,472.72)	511.40 ± 63.21 (474.91,547.89)	539.26 ± 44.57 (512.33,566.20)	503.04 ± 42.71 (478.38,527.70)	528.37 ± 38.21 (505.28,551.46)	488.95 ± 45.70 (462.57,515.34)	507.89 ± 46.09 (480.04,535.74)
Percentage of achieving 8-h sleep (%)	27.03	26.71	62.25	96.15	66.39	80.24	64.91	71.03
Physical activity parameters.								
Step count (steps)	12141.88 ± 3721.20 (9993.32,14290.44)	9270.66 ± 1223.86* (8531.09,10010.23)	9420.71 ± 5763.31 (6093.08,12748.35)	4612.23 ± 2652.36* (3009.43,6215.04)	8812.57 ± 2700.94 (7253.09,10372.04)	5209.01 ± 1920.26* (4048.61,6369.41)	7722.62 ± 2840.79 (6082.40,9362.84)	5023.56 ± 2279.31* (3646.19,6400.93)
MVPA (min)	56.93 ± 20.49 (45.09,68.76)	43.90 ± 14.35 (35.23,52.57)	37.46 ± 45.22 (11.36,63.57)	8.46 ± 9.14* (2.94,13.99)	33.91 ± 15.32 (25.07,42.76)	20.29 ± 15.15* (11.14,29.44)	30.21 ± 11.15 (23.77,36.65)	29.32 ± 24.79 (14.34,44.30)
Proportion of MVPA reaching 60 min (%)	40.63	27.52	20.83	3.83	19.37	10.77	14.84	14.98
Continuous MVPA (min)	31.04 ± 16.31 (21.63,40.46)	18.59 ± 7.56* (14.02,23.16)	20.46 ± 38.26 (−1.63,42.56)	2.42 ± 8.74 (−2.86,7.70)	16.25 ± 14.07 (8.13,24.37)	5.71 ± 4.42* (3.04,8.38)	12.78 ± 6.34 (9.11,16.44)	10.93 ± 10.60 (4.52,17.34)
Proportion of continuous MVPA in total MVPA time	0.40 ± 0.10 (0.33,0.46)	0.31 ± 0.12* (0.23,0.38)	0.19 ± 0.30 (0.02,0.37)	0.04 ± 0.13 (−0.04,0.12)	0.23 ± 0.16 (0.14,0.32)	0.10 ± 0.07* (0.06,0.14)	0.22 ± 0.09 (0.17,0.26)	0.13 ± 0.12 (0.06,0.21)

Data are presented as Mean ± SD (95% CI); * indicates a significant difference between genders at the same stage, *P* < 0.05.

## 4 Discussion

This study conducts a longitudinal investigation using wearable devices on middle school students in developed regions of Eastern China, revealing the differences in sleep and physical activity patterns across four phases: school days, weekends, winter vacation, and summer vacation. The findings provide empirical support for the ‘Structured Days Hypothesis’ within the context of China’s high academic pressure environment and further demonstrate that the highly structured school environment sustains certain levels of physical activity while exacerbating sleep deprivation. During relatively unstructured vacation periods, although sleep shows partial improvement, physical activity exhibits a ‘cliff-like’ decline. This indicates that in high-academic-pressure environments, optimizing adolescent behavioral patterns faces the dilemma of balancing sleep and physical activity. This study will delve into the changes in sleep and physical activity, seasonal differences during winter and summer vacations, and the potential mechanisms of gender differentiation, providing a more targeted theoretical basis and practical insights for the health of adolescents in developed cities in eastern China.

The study validated the overall pattern of “insufficient sleep on school days with relatively higher activity levels, while improved sleep but decreased activity during holidays.” This finding not only fundamentally aligns with the Structured Day Hypothesis (SDH) (19), but also corresponds with previous research regarding reduced physical activity and delayed sleep–wake patterns during holidays (40). On school days, specific physical activities such as commuting, physical education classes, and recess provide some level of physical activity; however, only 34.30% of students meet the MVPA (Moderate to Vigorous Physical Activity) standard of ≥60 min per day, which is significantly below the recommended health level. Furthermore, mandatory early school arrival and high academic workloads exacerbate sleep deprivation (41). Resulting in only 26.71% of students meeting sleep requirements, reflecting a state of “dual deficiency in both sleep and activity.” During holidays, the removal of external constraints leads to improved sleep quality, particularly with increased durations of deep sleep and REM sleep, indicating a partial restoration of physiological and emotional functions (42–44). However, physical activity experiences a dramatic decline, with extremely low MVPA compliance rates—only 3.83% among girls on weekend days—resulting in a “fragmented” holiday pattern that is highly prone to inducing obesity and metabolic risks (45). In China, parental expectations, children’s homework burdens, and increased media accessibility all contribute to elevated sedentary behavior (41, 46). In the context of China’s “Double Reduction” policy, the reduction of schoolwork burden and academic pressure does not necessarily lead to an increase in high-quality physical activity among students. Without collaborative planning and active guidance from families, communities, and schools, holiday periods are likely to be dominated by screen-based entertainment and sedentary activities, resulting in a “high-sedentary—low-activity” environment (47, 48). The findings of this study indicate that holidays are not inherently less healthy; rather, they highlight that in the context of China’s intense academic pressure, relying solely on the structured days of school schedules or depending entirely on the natural sleep improvements during holidays cannot achieve a dual optimization of both sleep and physical activity. While school days provide necessary structure, they often compromise sleep and still lack sufficient guarantees for physical activity. Conversely, holidays may enhance sleep but often result in a decrease in physical activity levels. Furthermore, research suggests that excessive disparities between school-day and holiday routines can lead to circadian rhythm misalignment and increase the risk of metabolic obesity (49, 50). Therefore, it is essential to implement efforts at both policy and practice levels. During school days, priority should be given to ensuring adequate sleep duration and enhancing the quality of physical activities. During holidays, a sustainable physical activity environment should be established through family support, community resources, and school coordination to prevent a significant decline in activity levels.

This study found that total sleep duration, deep sleep duration, and daily step counts during winter vacation were significantly higher than those during summer vacation. This difference may be influenced by both seasonal climatic factors and cultural festival elements. From a seasonal perspective, shorter daylight hours and lower temperatures in winter promote prolonged sleep duration, whereas summer’s high temperatures and intense sunlight may diminish both sleep duration and quality (51–53). Additionally, longer daylight hours in summer may disrupt circadian rhythms and affect normal melatonin secretion (54). Regarding physical activity, the increased step counts during winter vacation might be associated with frequent social visits and outings during the Spring Festival (55), while the hot weather in summer often discourages children’s engagement in outdoor activities (56). Despite the winter vacation outperforming the summer vacation across multiple indicators, the proportion of students meeting the recommended standards for moderate-to-vigorous physical activity (MVPA) remains low in both periods—15.01% during winter vacation and 14.91% during summer vacation—indicating overall unsatisfactory activity levels. This suggests that holiday health promotion strategies should be tailored to seasonal characteristics. During winter vacations, traditional festivals can be leveraged to encourage family outdoor interactions, transforming social activities into opportunities for physical activity. For summer vacations, providing indoor exercise guidelines and organizing summer camps could help mitigate the limiting effects of high temperatures on physical activities. The interpretations related to climate, sunlight, and festive activities in this study are based on potential mechanisms proposed in previous literature (52, 53), rather than direct evidence from this research. Future studies could integrate wearable devices, environmental monitoring data, and holiday behavior logs to comprehensively consider multiple factors such as climate, culture, and holiday structure, in order to further examine the specific sources of differences in sleep and physical activity across different holiday phases.

The allocation of time between sleep and physical activity exhibits significant gender differences. During the same developmental stage, female adolescents demonstrate longer durations of deep sleep, which may be associated with their earlier onset of growth hormone secretion patterns (57). Females experience an earlier rise in growth hormone secretion rates compared to males during puberty (58). On weekend days, female adolescents exhibit more pronounced sleep–wake phase delays than their male counterparts. Research indicates that social jetlag in females has a stronger association with depressive symptoms (59, 60). Therefore, it is crucial to pay particular attention to sleep–wake rhythm disruptions during holidays among female adolescents and their potential emotional impacts. In terms of physical activity, boys consistently demonstrate higher activity levels than girls across all age groups, which aligns with the global observation of generally lower moderate-to-vigorous physical activity among adolescent girls (61). This disparity arises not only from boys’ greater inclination toward high-intensity activities and girls’ lower participation in organized sports (62), but may also reflect the tendency for girls’ exercise opportunities to be supplanted by sedentary behaviors and screen time when they are removed from structured school environments (63). Importantly, this gap cannot be solely attributed to physiological factors or personal preferences; greater attention should be directed toward the potential influences of societal gender roles and family upbringing concepts. Traditional Chinese cultural expectations for girls to be gentle and quiet, or well-behaved, combined with families’ heightened safety concerns regarding girls’ outdoor activities, may restrict their opportunities and willingness to engage in autonomous, high-intensity activities during unstructured time (64–66). International longitudinal studies also indicate that changes in physical activity from adolescence to young adulthood exhibit significant gender characteristics (67, 68). It is important to emphasize that this study did not directly measure factors such as gender role cognition. Therefore, the interpretation regarding the influence of socio-cultural factors on girls’ physical activity should be considered a potential mechanism that requires further validation in future research. To promote activity among girls, it is essential to break down gender stereotypes and design more social and engaging forms of activities, such as dance and team games. Additionally, enhancing participation motivation through parental involvement and peer invitations can create a more friendly and supportive activity environment for them.

This study has several limitations. First, the sample size was relatively small, and all participants were drawn from the same school, which limits the generalizability of the findings. Future studies could utilize multicenter sampling to enhance regional diversity. Second, this research only conducted 1 year of longitudinal tracking, which fails to encompass a longer time span; this limitation makes it challenging to identify the changing trends of sleep and physical activity as individuals age. Third, the study did not simultaneously collect potential confounding factors such as screen time, socioeconomic status, academic workload, extracurricular activities, dietary habits, mental health status, and parental supervision. Finally, future research could further incorporate data from the spring semester to verify the consistency of behavioral patterns across different academic terms and to explore potential differences.

## 5 Conclusion

This study is based on longitudinal monitoring data from a small sample of students at a junior high school in Nanjing. It was initially found that students experience insufficient sleep on school days, while their physical activity levels are relatively high. Although sleep improves during holidays, overall physical activity declines. The winter vacation shows better results in both sleep duration and step count compared to the summer vacation. Girls have longer deep sleep durations than boys, but their physical activity levels are lower. These results indicate that, in the context of high academic pressure in developed regions in eastern China, relying solely on school structure or natural improvements during holidays cannot achieve a dual optimization of sleep and activity. Systematic environmental and behavioral interventions are necessary. It is recommended to prioritize sleep time and optimize daily schedules during school days; during holidays, structured physical activity opportunities should be provided through collaboration among families, schools, and communities, such as school sports assignments, parent–child exercise, and community sports activities. Additionally, gender and seasonal differences should be considered, designing more appealing exercise programs for girls, and promoting indoor or community exercise guidance during the summer vacation to facilitate phased and gender-specific improvements in health behaviors.

## Data Availability

The raw data supporting the conclusions of this article will be made available by the authors, without undue reservation.
